# A novel immortalized hepatocyte-like cell line (imHC) supports in vitro liver stage development of the human malarial parasite *Plasmodium vivax*

**DOI:** 10.1186/s12936-018-2198-4

**Published:** 2018-01-25

**Authors:** Yongyut Pewkliang, Siriwan Rungin, Kaewta Lerdpanyangam, Apisak Duangmanee, Phongthon Kanjanasirirat, Phichaya Suthivanich, Khanit Sa-ngiamsuntorn, Suparerk Borwornpinyo, Jetsumon Sattabongkot, Rapatbhorn Patrapuvich, Suradej Hongeng

**Affiliations:** 1 Excellent Center for Drug Discovery (ECDD), Faculty of Science, Mahidol University, Bangkok, Thailand; 2 Mahidol Vivax Research Unit, Faculty of Tropical Medicine, Mahidol University, Bangkok, Thailand; 3 Department of Biochemistry, Faculty of Pharmacy, Mahidol University, Bangkok, Thailand; 4 Department of Biotechnology, Faculty of Science, Mahidol University, Bangkok, Thailand; 5 Center for Emerging and Neglected Infectious Diseases, Mahidol University, Bangkok, Thailand; 6 Department of Pediatrics, Faculty of Medicine Ramathibodi Hospital, Mahidol University, Bangkok, Thailand; 7 Present Address: Drug Research Unit for Malaria, Faculty of Tropical Medicine, Mahidol University, Bangkok, Thailand; 8 Present Address: Siriraj Initiative in System Pharmacology, Faculty of Medicine Siriraj Hospital, Mahidol University, Bangkok, Thailand

**Keywords:** Malaria, *Plasmodium vivax*, Sporozoite, Liver stage, imHCs

## Abstract

**Background:**

Eradication of malaria is difficult because of the ability of hypnozoite, the dormant liver-stage form of *Plasmodium vivax*, to cause relapse in patients. Research efforts to better understand the biology of *P*. *vivax* hypnozoite and design relapse prevention strategies have been hampered by the lack of a robust and reliable model for in vitro culture of liver-stage parasites. Although the HC-04 hepatoma cell line is used for culturing liver-stage forms of *Plasmodium*, these cells proliferate unrestrictedly and detach from the culture dish after several days, which limits their usefulness in a long-term hypnozoite assay.

**Methods:**

A novel immortalized hepatocyte-like cell line (imHC) was evaluated for the capability to support *P. vivax* sporozoite infection. First, expression of basic hepatocyte markers and all major malaria sporozoite-associated host receptors in imHC was investigated. Next, in vitro hepatocyte infectivity and intracellular development of sporozoites in imHC were determined using an indirect immunofluorescence assay. Cytochrome P450 isotype activity was also measured to determine the ability of imHC to metabolize drugs. Finally, the anti-liver-stage agent primaquine was used to test this model for a drug sensitivity assay.

**Results:**

imHCs maintained major hepatic functions and expressed the essential factors CD81, SR-BI and EphA2, which are required for host entry and development of the parasite in the liver. imHCs could be maintained long-term in a monolayer without overgrowth and thus served as a good, supportive substrate for the invasion and growth of *P. vivax* liver stages, including hypnozoites. The observed high drug metabolism activity and potent responses in liver-stage parasites to primaquine highlight the potential use of this imHC model for antimalarial drug screening.

**Conclusions:**

imHCs, which maintain a hepatocyte phenotype and drug-metabolizing enzyme expression, constitute an alternative host for in vitro *Plasmodium* liver-stage studies, particularly those addressing the biology of *P. vivax* hypnozoite. They potentially offer a novel, robust model for screening drugs against liver-stage parasites.

**Electronic supplementary material:**

The online version of this article (10.1186/s12936-018-2198-4) contains supplementary material, which is available to authorized users.

## Background

Malaria remains a major health problem worldwide, with an estimated 212 million disease cases and hundreds of thousands of related deaths in 2015 alone [1]. Malarial parasites are transmitted to humans via bites of infected female *Anopheles* mosquitoes; specifically, these parasites reside in the sporozoite form in mosquito salivary glands and are injected into human skin. The sporozoites rapidly leave the injection site and migrate through the bloodstream to the liver, where they invade hepatocytes and develop into liver-stage schizonts. A mature schizont contains thousands of daughter forms, termed merozoites, which are eventually released into the bloodstream where they invade erythrocytes and trigger the clinical symptoms of the disease. Of the five *Plasmodium* species that infect humans, *Plasmodium falciparum* is thought to cause the majority deaths, of which most cases (70%) involve children aged < 5 years in sub-Saharan Africa [1]. *Plasmodium vivax* is the most widespread *Plasmodium* species outside Africa. This organism is recognized as a major obstacle in malaria eradication campaigns because it can hide in a form called a hypnozoite [2, 3] in the human liver, and can reactivate weeks, months or years after the primary infection to cause a relapse [4–8].

The *Plasmodium* liver stage has become an attractive target for the development of anti-malarial drugs and vaccines because it is the precursor to blood-stage disease and also serves as a reservoir of the hypnozoites [9]. To date, only one approved drug, primaquine (PQ), has been shown to effectively eliminate liver-stage parasites, including *P. vivax* hypnozoites [10, 11]; however, this drug induces haemolytic toxicity in glucose-6-phosphate dehydrogenase-deficient individuals [12] and drug tolerance and therapeutic failures have compromised its clinical use [13–16]. Avoidance of these toxic effects have been considered in the development of an alternative drug, tafenoquine (TQ) [17, 18], which targets hypnozoites and is currently in Phase III clinical trials [19]. However, the development of more safe and efficacious anti-liver stage drugs, particularly those that target hypnozoites, remains essential [20].

The lack of a robust, reliable in vitro model of the *Plasmodium* liver stage has limited understanding of the biology of *Plasmodium* liver stage and consequently, hampered development of drug and discovery programmes [19]. Current in vitro models of human liver-stage parasites (mainly *P. falciparum and P. vivax*) use both hepatoma cell lines, such as HepG2-A16 and HC-04 [21–24], and primary human hepatocytes [25–28]. The major advantages of the hepatoma cell lines include reliability and reduced variability between infection batches. Nonetheless, these cells exhibit abnormal cell regulation and proliferation and thus do not accurately recapitulate the biology of a liver-stage infection. Primary human hepatocytes are considered an ideal model for liver-stage cultures because these are the natural hosts. Nevertheless, primary hepatocytes are rarely used because of their limited availability and gradual loss of hepatic functions over time under conventional culture conditions [29]. Several groups have attempted to establish models that could prolong functional phenotypes of primary hepatocytes in culture. The primary hepatocyte and human hepatoma HepaRG cell co-culture model has been shown to not only retain hepatic functions but also to help maintain the susceptibility of primary hepatocytes to *P. falciparum* infection [30]. Another advanced micropatterned co-culture (MPCC) model, in which primary hepatocytes are organized among supportive stromal cells (3T3-J2 fibroblast cells), has been shown to retain functional hepatocytes for up to 4–6 weeks [28, 31–34] and thus, allow the establishment of *P. falciparum* and *P. vivax* liver stage forms [28]. Recently, a microfluidic bilayer device was developed to promote long-term stability of primary hepatocytes and offer another platform for human liver-stage culture in vitro [35].

A ‘continuous non-tumorous cell line’ in which hepatocyte phenotypes are maintained could be a necessary substitute for primary hepatocytes. Some alternative sources of human cells that mimic the phenotypes of hepatocytes have been developed. In recent advances, induced pluripotent stem cell-derived hepatocyte-like cells (iHLCs) have been shown to support *Plasmodium* liver stages [36]. Nevertheless, these iHLCs are functionally immature and their hepatic functions must be induced and extensively characterized before each use [37]. Moreover, iHLCs that have been induced to differentiate usually exhibit limited life spans. Accordingly, iHLCs do not seem to be a simple or robust option for an in vitro model.

A novel ‘immortalized’ hepatocyte-like cell line (imHC) derived from human bone marrow mesenchymal stem cells (hMSCs) has been established [38]. These imHCs maintain the production of hepatocyte-specific markers, including albumin (ALB), urea, glycogen, alpha-fetoprotein (AFP), tyrosine aminotransferase, hepatocyte nuclear factor-4-alpha (HNF-4ɑ), glucose-6-phosphate dehydrogenase and all major cytochrome P450 isotypes (CYP450s). In this report, the feasibility of imHCs as a model for establishing a malaria infection was demonstrated. imHCs support the growth of *P. vivax* liver stages. This study also highlights the potential use of imHCs as a model for drug screening applications.

## Methods

### Ethical approval

In this study, human blood was collected and patient samples were used in strict accordance with the human use protocol TMEC 11-033, approved by the Institute Ethical Review Committee of the Faculty of Tropical Medicine, Mahidol University, Bangkok, Thailand. Written informed consent was obtained from the patient for the publication of this report.

### *Plasmodium* sporozoite preparation

Laboratory-reared female *Anopheles dirus* mosquitoes were maintained in a colony at the Mahidol Vivax Research Unit in Bangkok, Thailand. The mosquitoes were membrane-fed with blood samples collected from *Plasmodium*-infected patients in Kanchanaburi and Tak Provinces, Thailand. Briefly, 5 ml of patient blood was collected in a heparinized tube and centrifuged at 1500×*g* for 10 min to remove plasma. The resulting pellet was washed with 10 ml of phosphate-buffered saline (PBS) and reconstituted to the original volume with naïve-type AB serum for mosquito feeding [39]. Each feeder contained approximately 0.5 ml of infected blood and was used to feed approximately 100 5–7-day-old mosquitoes during a 30-min period. Mosquitoes were subsequently maintained on a 10% sucrose solution under a controlled environment at 26 °C and 75% humidity with a 12-h light/dark cycle. Midgut oocysts of *P. vivax* were monitored on dissected mosquitoes using a mercurochrome staining method on day 7 post feeding [40]. The salivary gland sporozoites were examined on day 14 post feeding and dissected from the infected mosquitoes using a standard protocol [41]. In brief, salivary glands of 50 infected mosquitoes were dissected, placed in 50 μl of ice-cold RPMI 1640 medium (Gibco, Grand Island, NY, USA), pH 8.2 [42], supplemented with 200 U/ml penicillin (Invitrogen, Carlsbad, CA, USA), 200 µg/ml streptomycin (Invitrogen), and 0.25 µg/ml amphotericin B (Invitrogen), and ground with a sterile pestle. The released sporozoites were counted in a hemocytometer and kept on ice until used, but for no more than 1 h to avoid a reduction in parasite infectivity (Patrapuvich R, unpublished data and [43]).

### Hepatocyte culture

imHCs were cultured in 1:1 DMEM:Ham’s F12 media (Invitrogen) supplemented with 10% heat-inactivated fetal bovine serum (FBS) (Gibco), 100 U/ml penicillin, and 100 µg/ml streptomycin. The HC-04 hepatoma cell line (ATCC patent deposit no. PTA-3441: ATCC, Manassas, VA, USA) was cultured in 1:1 minimal essential medium (MEM):Ham’s F12 media (Invitrogen) supplemented with 10% heat-inactivated FBS, 100 U/ml penicillin, and 100 µg/ml streptomycin. Both cell lines were maintained at 37 °C in a humidified atmosphere containing 5% CO_2_. Cells were subcultured every 2–4 days or once they had reached approximately 80% confluence, after detachment with 0.125% trypsin–EDTA (Invitrogen).

### Growth curve

Hepatocytes were seeded at a density of 2 × 10^4^ cells per well in a six-well plate (Thermo Scientific™, Nunc™; Waltham, MA, USA) and maintained as described above for 2 weeks with daily changes in medium. Viable cells were monitored and counted daily using a hemocytometer and an Olympus IX71 inverted phase/fluorescence microscope (Olympus, Tokyo, Japan). Growth curves were generated using GraphPad Prism software, version 7.0 (GraphPad Inc, La Jolla, CA, USA).

### In vitro liver-stage infection

Hepatocytes were seeded at a density of 3 × 10^5^ cells per well in a Matrigel (Corning Corp, Corning, NY, USA)-coated Millicell EZ SLIDE eight-well glass slide (Millipore, Billerica, MA, USA) and maintained in complete medium (1:1 MEM:Ham F12 supplemented with 10% FBS, 100 U/ml penicillin, and 100 µg/ml streptomycin) at 37 °C. After incubation for 18–20 h, the medium was aspirated, and an aliquot of sporozoite suspension (3 × 10^5^ in 200 µl of complete medium) was added to each well. After a 4-h inoculation at 37 °C, free sporozoites were removed by aspiration and a 400-µl aliquot of fresh infection medium (1:1 MEM:Ham F12 supplemented with 10% FBS, 100 U/ml of penicillin, 100 µg/ml of streptomycin, and 0.25 µg/ml amphotericin B) was added. The infected hepatocyte culture was maintained at 37 °C with daily changes in medium until the liver-stage parasites or exoerythocytic forms (EEs) were established and visualized using an indirect immunofluorescence assay. The total number of EEs in each well was manually quantified. Per cent sporozoite infectivity was determined by comparing the number of infected hepatocytes to the total number of inoculated sporozoites [42].

### Indirect immunofluorescence assay

After removing the culture medium, the infected hepatocyte cells were washed with PBS buffer thrice, fixed with 4% paraformaldehyde for 20 min, and permeabilized by exposure to 0.1% Triton X-100 for 3–5 min at room temperature (20–25 °C). The cells were then treated with 3% BSA (in PBS) solution, followed by incubation with mouse primary monoclonal anti-PbHSP70 antibodies (clone 4C9, kindly provided by Dr. Fidel P. Zavala [44]) and rabbit primary polyclonal anti-PvUIS4 antibodies (1:500 dilution, kindly provided by Dr. Sebastian A. Mikolajczak [45]). Subsequently, goat secondary IgG Alexa Fluor^®^ 488-conjugated anti-mouse antibodies (1:500 dilution, Invitrogen), goat secondary IgG Alexa Fluor^®^ 568-conjugated anti-rabbit 2nd antibodies (1:500 dilution, Invitrogen) and 0.1 μg/ml 4′,6-diamidino-2-phenylindole (DAPI) (Invitrogen) were added to the cells. Incubation with primary and secondary antibodies was performed at 37 °C for 1 h and 30 min, respectively. In some experiments, rabbit anti-acetylated histone H3K9 antibodies (1:200 dilution, Millipore) and mouse anti-acyl carrier protein (ACP) antibodies (1:200 dilution, kindly provided by Dr. Sebastian A. Mikolajczak [45]) were used for detecting nuclear histones and apicoplasts of *P. vivax* EEs, respectively. Finally, samples were covered with ProLong Gold antifade reagent (Invitrogen), sealed under coverslips, and viewed under a fluorescent microscope (400× magnification; AXIO Scope. A1 equipped with AxioVision Rel 4.8 Software; Carl Zeiss AG, Oberkochen, Germany). Fluorescence images were acquired using an Olympus FluoView™ FV1000 confocal laser scanning microscope (Olympus, Tokyo, Japan) equipped with a 60× oil objective. Images were captured using FV10-ASW 3.0 viewer software and prepared for publication using Adobe Illustrator CC (Adobe Systems, San Jose, CA, USA).

In some experiments, uninfected hepatocyte cells were grown in 96-well CellCarrier-96 optic black plates (PerkinElmer, Waltham, MA, USA) and stained with antibodies against the following hepatocyte markers: ALB (1:100 dilution, ab10241, Abcam, Cambridge, UK), AFP (1:100 dilution, SC8399, Santa Cruz Biotechnology, Dallas, TX, USA), low-density lipoprotein receptor (LDLR; 1:100 dilution, SC373830, Santa Cruz Biotechnology), Na^+^-taurocholate cotransporting polypeptide (NTCP; 1:100 dilution, ab131084, Abcam, Cambridge, UK), and HNF-4ɑ (1:100 dilution, SC6556, Santa Cruz Biotechnology). Cells were also stained with antibodies against the following cell surface receptors: CD81 (1:100 dilution, ab79559, Abcam), scavenger receptor type B class I (SR-BI) (1:100 dilution, NB400-104, Novus Biologicals, Littleton, CO, USA), and EphA2 (1:100 dilution, 37-4400, Thermo Fisher Scientific). Samples were then incubated with a goat anti-mouse Alexa Fluor^®^ 488-conjugated (1:500 dilution, Invitrogen), goat anti-rabbit Alexa Fluor^®^ 488-conjugated (1:500 dilution, Invitrogen), or donkey anti-goat Cy3-conjugated secondary antibody (1:500 dilution, BioLegend, San Diego, CA, USA), as appropriate; hepatocyte nuclei were stained with 2 µM Hoechst 33342 (Thermo Fisher Scientific). Mouse IgG2a, mouse IgG1, rabbit IgG and goat IgG were used as negative control for staining. Fluorescence images were captured and analysed using an Operetta High-Content Imaging System (PerkinElmer) with a 40× objective lens.

### Primaquine treatment

PQ (Sigma-Aldrich, St Louis, MO, USA) was added to infected hepatocytes at concentrations ranging from 0.1 to 10 μM 2 h post sporozoite infection. The cultures were maintained for up to 5 days with daily changes in medium (including drug supplementation) and harvested on day 6 to determine sporozoite infectivity by immunofluorescence assay, as described above. Numbers and sizes of EEs were manually examined using a fluorescent microscope (ZEISS AXIO Scope.A1). The relative proportions of small EEs (diameter ≤ 5 µm) and schizonts (diameter > 5 µm) were determined relative to the 0.1% dimethyl sulfoxide untreated controls. *Plasmodium vivax* assays were conducted over three independent experiments using three batches of sporozoites generated from different *P. vivax* isolates.

### Quantitative real-time PCR

Total RNA was extracted from the hepatocytes using a RNeasy Plus Mini Kit (Qiagen, Hilden, Germany). The isolated total RNA was then treated with DNase I (Thermo Scientific, USA), according to the manufacturer’s instructions. The quantity and quality of total RNA were determined using a NanoDrop spectrophotometer (Thermo Scientific). For the CYP450 qRT-PCR analysis, 500 ng of total RNA was converted to cDNA using the SuperScript^®^ III First-Strand Synthesis System (Invitrogen). The primer sets and conditions used for CYP450 amplification have been described previously [38]. Briefly, 2 μl of cDNA was diluted in a 10-μl reaction mixture containing 0.4 μM of each primer and 5 μl of Luminaris Color HiGreen qPCR Master Mix (Thermo Scientific, USA). Each reaction was run in a CFX96 Touch™ Real-Time PCR Detector (Bio-Rad, Hercules, CA, USA) with the following conditions: 50 °C for 2 min; 95 °C for 10 min; and 40 cycles of amplification at 95 °C for 15 s and 60 °C for 45 s. Reverse transcriptase- and template-negative controls were used for each gene-specific primer pair. The number of cycles required for the fluorescent signal to cross the threshold (Ct value) was determined for each CYP450 isotype. Obtained Ct values were subtracted from Ct of the respective house-keeping gene (GAPDH) obtained from the same cells to generate a relative gene expression value ΔCt. In some experiments, hepatocytes were treated with PQ for 6 days with daily changes in medium prior to RNA extraction.

### Statistical analysis

At least three independent experiments were conducted in triplicate. Each individual sporozoite infection experiment was performed using a different parasite isolate. Differences in the results were determined using a standard Student’s *t* test, and a *p* value of < 0.05 was considered to indicate statistical significance. For non-normally distributed data, the Mann–Whitney *U* test was used in place of the paired *t* test.

## Results

### imHCs expressed hepatic phenotypes

**Fig. 1:**
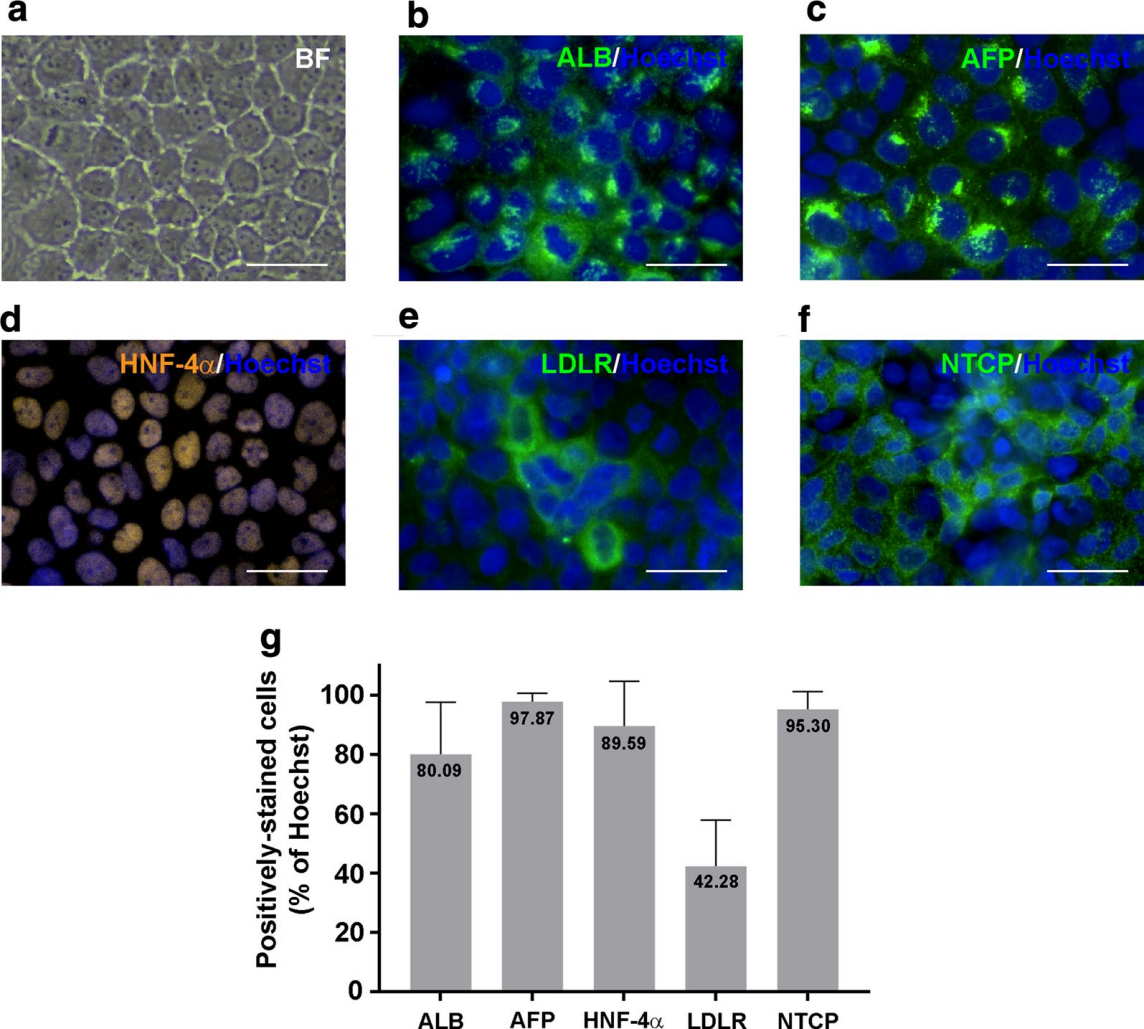
Characterization of immortalized hepatocyte-like cells (imHCs). imHCs were maintained in DMEM/F12 medium containing 10% fetal bovine serum. After reaching confluence, cells exhibited a typical hepatocyte morphology, including a polygonal shape, granulated cytoplasm, and large nucleus (**a**). Hepatocyte phenotypes were evaluated via immunofluorescence staining for the following major hepatocyte markers: albumin (ALB) (**b**), ɑ-fetoprotein (AFP) (**c**), hepatocyte nuclear factor-4-alpha (HNF-4ɑ) (**d**), low-density lipoprotein receptor (LDLR) (**e**), and Na^+^-taurocholate cotransporting polypeptide (NTCP) (**f**). Cell nuclei were visualized using Hoechst 33342 DNA dye. Fluorescence images were captured and analysed using an Operetta High-Content Imaging System (PerkinElmer) with a ×40 objective lens. Scale bar = 50 μm. The presence of hepatic marker in imHCs was quantified from 15 randomly selected image fields (total number of analysed cells > 2000) (**g**). Columns represent the percentage of cells containing ALB, AFP, HNF-4ɑ, LDLR or NTCP. Data are represented as mean ± standard deviations

hMSCs, precursors of imHCs, were immortalized via lentiviral transduction with the human telomerase reverse transcriptase gene (*hTERT*) and *Bmi*-*1* [46], according to previously described protocol [38]. After induction of differentiation, resulting imHCs were examined for hepatocyte phenotype and metabolic function maintenance, and for continuous cell line property using population doubling level (PDL) [38]. imHCs exhibited a typical homogenous hepatocyte morphology, polygonal shape, and cord-like structure (Fig. 1a), and expressed basic hepatocyte-specific markers, including ALB, AFP, HNF-4ɑ, LDLR, and NTCP, as demonstrated by immunofluorescence staining (Fig. 1b–f, respectively). As shown in Fig. 1g, more than 80% of the population of imHCs contained ALB, AFP, HNF-4ɑ, and NTCP and approximately 42% of the cells appeared positive for LDLR. The expression level of LDLR in imHCs was comparable with that observed in human hepatocyte cell line HepG2 [47]. The expression of HNF-4ɑ, a hepatic maturation marker, indicated that imHCs had fully matured into hepatocytes. This result was consistent with the previous study, which also showed that the expression levels of the hepatic marker in imHCs were comparable to those in primary hepatocytes [38].

### imHCs expressed essential receptors for malaria infection

**Fig. 2:**
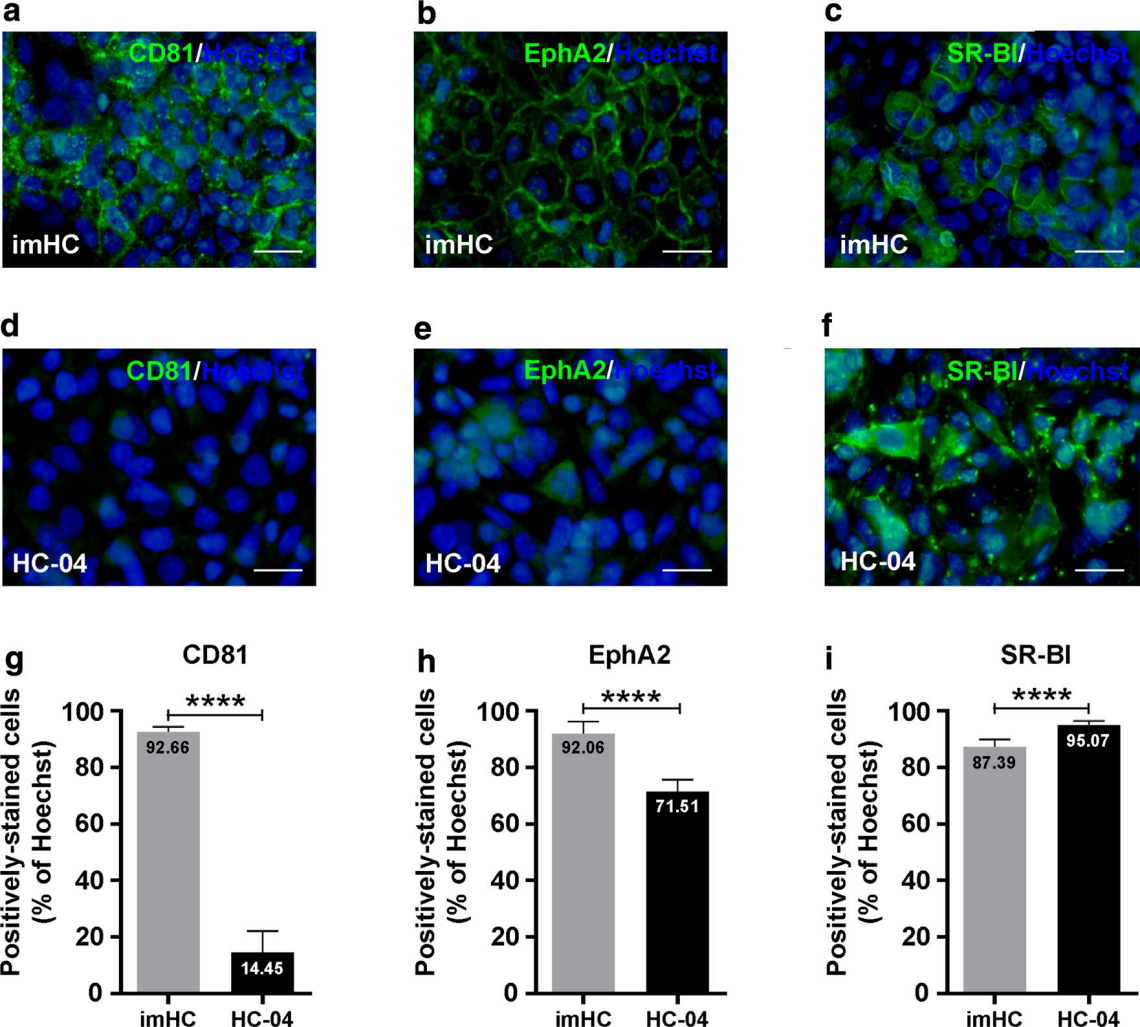
Identification of *Plasmodium* sporozoite-associated receptors on immortalized hepatocyte-like cells (imHCs). The expression of *Plasmodium* sporozoite-associated receptors (CD81, EphA2 and SR-BI) on imHCs and HC-04 cells was determined using immunofluorescent staining. Prior to analysis, the hepatocytes were grown in MEM/F12 medium containing 10% fetal bovine serum until confluence was reached. The expression of CD81 (**a**), EphA2 (**b**) and SR-BI (**c**) on imHCs and HC-04 cells was compared (**d**, **e**, **f**, respectively). Hepatocyte nuclei were stained with Hoechst 33342 dye. Fluorescence images were captured and analysed using an Operetta High-Content Imaging System (PerkinElmer) with a ×40 objective lens. Scale bar = 50 μm. Cells expressing CD81, EphA2 or SR-BI were quantified from 15 randomly selected image fields (total number of analysed cells > 2000) (**g**, **h**, **i**, respectively). Bar graph shows the mean percentage of positively stained cells. Error bars depict standard deviations. ****p < 0.0001, Student’s *t* test

To investigate the potential use of imHCs in malaria infection studies, the expression of essential molecules required for *Plasmodium* liver-stage infection on the putative host cell surfaces was examined. imHCs expressed all major malaria sporozoite-associated receptors known to be essential for parasite entry and intracellular development in the liver, including TAPA-1 (CD81) [48] (Fig. 2a), EphA2 [49] (Fig. 2b) and SR-BI [50] (Fig. 2c). CD81 and EphA2 were expressed at higher levels in imHCs than in HC-04 cells, a cell line most commonly used for malaria sporozoite infection (Fig. 2d, e). The numbers of CD81- and EphA2-positive cells were significant higher in imHCs than in HC-04 (Fig. 2g, h; *p* < 0.0001 for both cases). HC-04 cells exhibited clear inter-cellular variations in levels of EphA2, similar to the findings reported from hepatoma Hepa1-6 cultures [49]. Both imHCs and HC-04 cells expressed SR-BI at similar levels, with 87 ± 2.5% and 95 ± 1.4% of cells, respectively (Fig. 2i), although the protein localization differed slightly between the cells (Fig. 2c, f). Given these findings, imHCs could possibly be infected by *Plasmodium* malarial parasites and may be superior to the classical host (HC-04 cells).

### imHCs were suitable for long-term culture

**Fig. 3:**
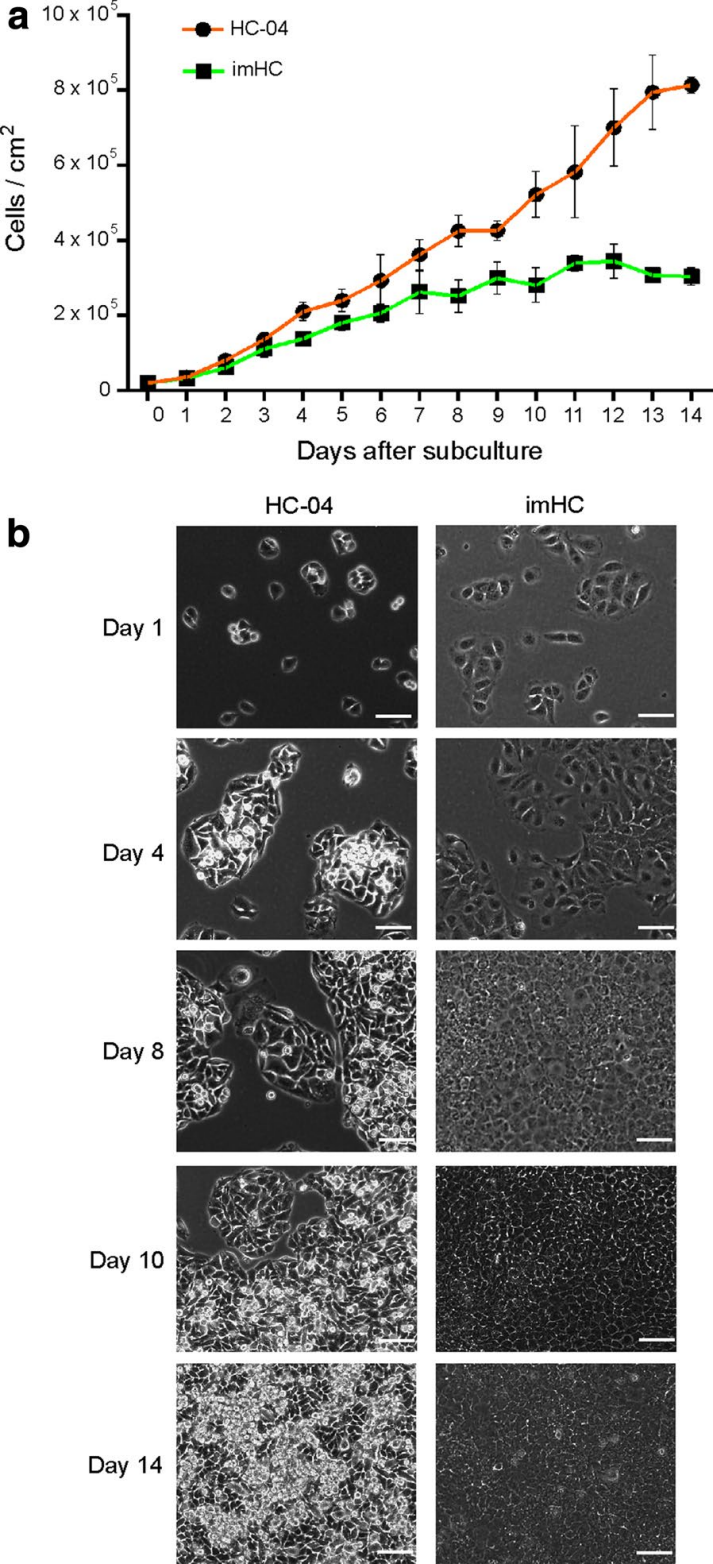
Cessation of immortalized hepatocyte-like cell (imHC) proliferation upon reaching confluency. The numbers of hepatocytes maintained in MEM/F12 medium containing 10% fetal bovine serum were recorded for more than 2 weeks to generate a standard growth curve. This experiment was performed in triplicate, and cell densities are presented as means ± standard deviations. imHCs exhibited a slower growth pattern than the widely used HC-04 hepatoma cell line (**a**) and stopped growing upon reaching confluency. Representative images of imHCs and HC-04 cells at days 1, 4, 8, 10, and 14 after subculture (**b**). HC-04 cells did not stop growing and formed multilayers of cells during a long-term culture, whereas imHCs maintained monolayer growth. Scale bar = 50 μm

imHCs grew more slowly than HC-04 cells, as demonstrated by their doubling times (Fig. 3a). The mean doubling time of imHCs was 45 h versus 40 h of HC-04 cells (Fig. 3a). The batch of HC-04 cells used in this study had a similar growth rate to the reported value, with a doubling time of approximately 37 h [23]. HC-04 cells were round in shape and tended to aggregate and clump together (Fig. 3b). While a reduction in HC-04 cell growth once the culture reached confluency was observed, HC-04 cells exhibited non-stop growth and a loss of cell–cell contact inhibition and formed a multi-layer arrangement of cells (Fig. 3b) that gradually detached from the culture plate after 3 weeks of culture. In contrast, imHCs displayed an epithelial-like morphology and clearly exhibited contact inhibition after reaching confluence (Fig. 3b). This property allowed imHC cells to remain in a monolayer without overgrowth for up to 6 weeks of culture (Additional file 1: Figure S1). This finding suggested that imHCs may be suitable for long-term culture of *P. vivax* hypnozoites. In addition, the flat imHC monolayer may not only help in prolonging the culture but also in improving the ability to visualize and detect liver-stage parasites using immunofluorescence microscopy.

### imHCs supported *Plasmodium vivax* liver-stage infection in vitro

**Fig. 4:**
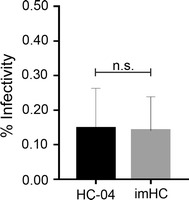
*Plasmodium vivax* infection in HC-04 cell and immortalized hepatocyte-like cell (imHC) cultures. Hepatocytes were grown in an eight-well plate at a density of 3 × 10^5^/well. Each well was infected with 3 × 10^5^ salivary gland *P. vivax* sporozoites. Infected hepatocytes were harvested, and the exoerythocytic forms were counted in an indirect immunofluorescence assay on day 4 post infection. The per cent sporozoite infectivity was determined by comparing the number of infected hepatocytes to the total number of inoculated sporozoites. All data are shown as means ± standard errors of the means of three independent experiments from three different *P. vivax* isolates. The Mann–Whitney *U* test was used to evaluate the *p* value. *ns* non-significant (*p* > 0.05)

Next, the feasibility of using imHCs for *P. vivax* infection was examined. First, imHCs were infected with *P. vivax* sporozoites and EEs were determined by immunostaining with an antibody specific for *Plasmodium* HSP70. EEs were evaluated on day 4 post sporozoite infection because this is the earliest day when the developing schizonts and small EEs of *P. vivax* could be differentiated. Consistent with the hypothesis, imHCs could be infected by *P. vivax* sporozoites. The total number of *P. vivax* EEs obtained from 3 × 10^5^ inoculated sporozoites was in the range of 30–1000, and this number did not significantly differ from the number observed in the HC-04 culture (*p* > 0.05). Infection efficiencies of the sporozoites were 0.14 ± 0.16% and 0.15 ± 0.19% in imHCs and HC-04 cells, respectively (Fig. 4).

**Fig. 5:**
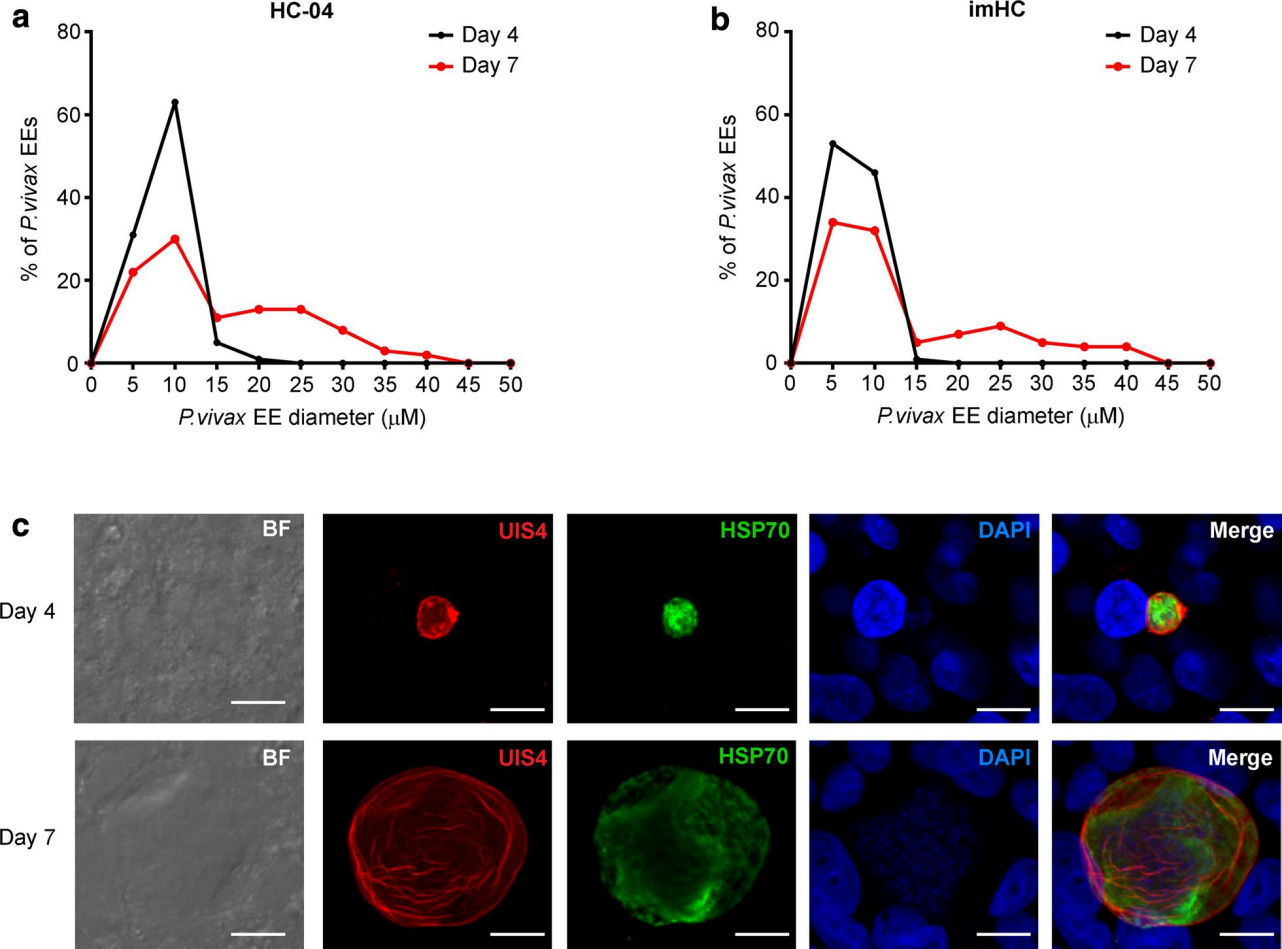
Immortalized hepatocyte-like cells (imHCs) were susceptible to *Plasmodium vivax* liver-stage infection. Size distributions of *P. vivax* exoerythocytic forms (EEs) on days 4 and 7 post-infection in HC-04 cells (**a**) and imHCs (**b**). Representative immunofluorescence images of the intracellular *P. vivax* EEs on days 4 and 7 post-infection (**c**). *Plasmodium vivax* EEs in imHCs were visualized using an indirect immunofluorescence assay with antibodies against parasite UIS4 (red) and HSP70 (green). The nuclei were counter-stained with DAPI (blue). Scale bar = 10 μm

To investigate whether imHCs could fully support further development of *P. vivax* sporozoites, the rate at which parasites developed into schizont forms (diameter > 10 μm) between days 4 and 7 post infection was monitored. Although imHCs and HC-04 cells were infected by different batches of sporozoites, similar patterns of development of EEs in these cells were observed (Fig. 5a, b). All EEs obtained during the 4-day period measured < 15 μm in diameter; however, on day 7, approximately 40% of EEs had progressed to the schizont stage (> 10 μm in diameter) of the liver-stage life cycle, with approximate maximum sizes of 40–45 μm (Fig. 5a, b). However, imHCs allow a better *Plasmodium* liver stage than HC-04 cells because larger EEs were found in the former (30–45 μm) relative to the latter (20–25 μm). Representative immunofluorescence images of *P. vivax* EEs on days 4 and 7 post sporozoite infection are shown in Fig. 5c and Additional file 2: Figure S2. The normal growth of *P. vivax* EEs in the imHC model was confirmed by the expression of parasitophorous vacuole membrane (PVM)-localized PvUIS4, a known liver-stage-specific marker [45]. Importantly, day 4 EEs exhibited a PvUIS4-positive PVM feature known as a ‘prominence’, which has been reported as a potential indicator of hypnozoites [45] (Fig. 5c, upper panel). Furthermore, small forms (< 10 μm) were also observed in imHCs after day 7 post-infection (Fig. 5a, b and Additional file 2: Figure S2).

**Fig. 6:**
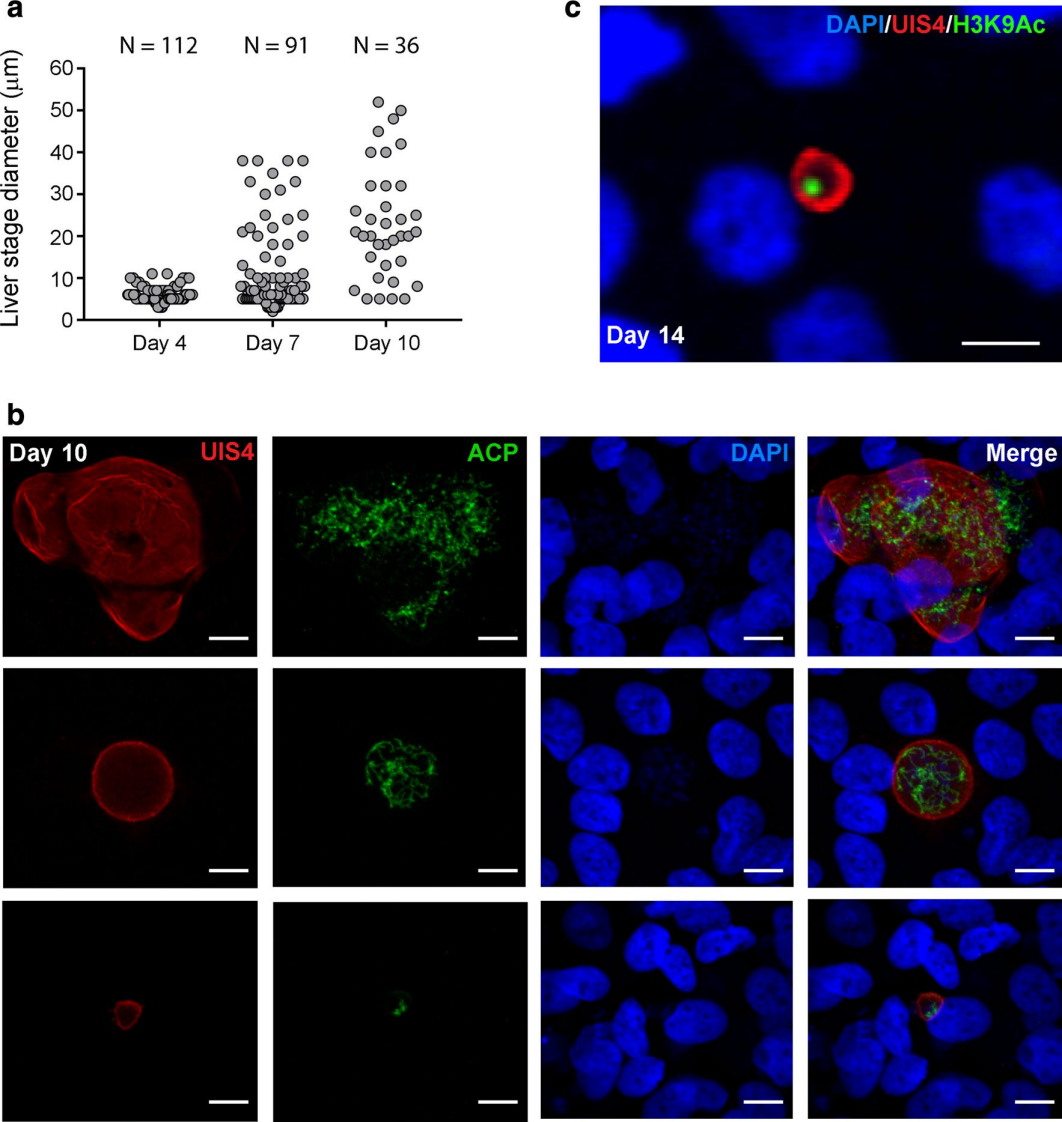
In vitro growth of intra-hepatocytic *Plasmodium vivax* in immortalized hepatocyte-like cell (imHC) culture. *Plasmodium vivax* sporozoites were co-cultured with imHCs for 4, 7, and 10 days. Exoerythocytic forms (EEs) were visualized in an indirect immunofluorescence assay using antibodies against parasite UIS4, ACP and H3K9Ac. The hepatocyte nuclei were stained using DAPI. The sizes of individual EEs from duplicate wells were measured at each time point and plotted (**a**), and representative immunofluorescence images of intracellular *P. vivax* EEs on day 10 (**b**) and 14 post-infection (**c**) are shown. Scale bar = 10 μm

Then, the maturation of *P. vivax* EEs in an imHC culture was monitored. EEs increased in size from an average of 5 μm on day 4 to a wide range of sizes up to 50 μm by day 10 post-infection (Fig. 6a, b and Additional file 3: Figure S3). The gradual decline in the numbers of EEs after day 10 was possibly attributable to the rupture of some mature schizonts and release of merozoites, indicating the completion of its life cycle in the liver (Fig. 6a). Asynchronous development is a normal characteristic of in vitro EE culture [23, 51] and was clearly observed in *P. vivax*-infected imHCs after day 7 (Fig. 6a). Representative images of day 10 EEs and asynchronous features of EEs, which ranged in size from < 10 μm to up to 50 μm, are shown in Fig. 6b. In imHCs, *P. vivax* EEs also displayed another typical liver-stage-specific marker, apicoplast-localized ACP [45], during a 10-day infection period, thus confirming the normal development of *P. vivax* in these cells (Fig. 6b). The presence of acetylated lysine 9 of histone H3-positive small EEs on day 14 post-infection (Fig. 6c) might indicate the existence of the hypnozoites in the culture; however, these small EEs in imHCs require further characterization. Moreover, two variants of Thai *P. vivax* sporozoites with the genotypes CSP-VK210 and CSP-VK247 were used in this study, and imHCs were found to be susceptible to infection by both variants. On day 7 post infection, the CSP-VK210 and CSP-VK247 variants yielded a small form frequency of approximately 36 and 77%, respectively.

### *Plasmodium*-infected imHCs were sensitive to PQ treatment

**Fig. 7:**
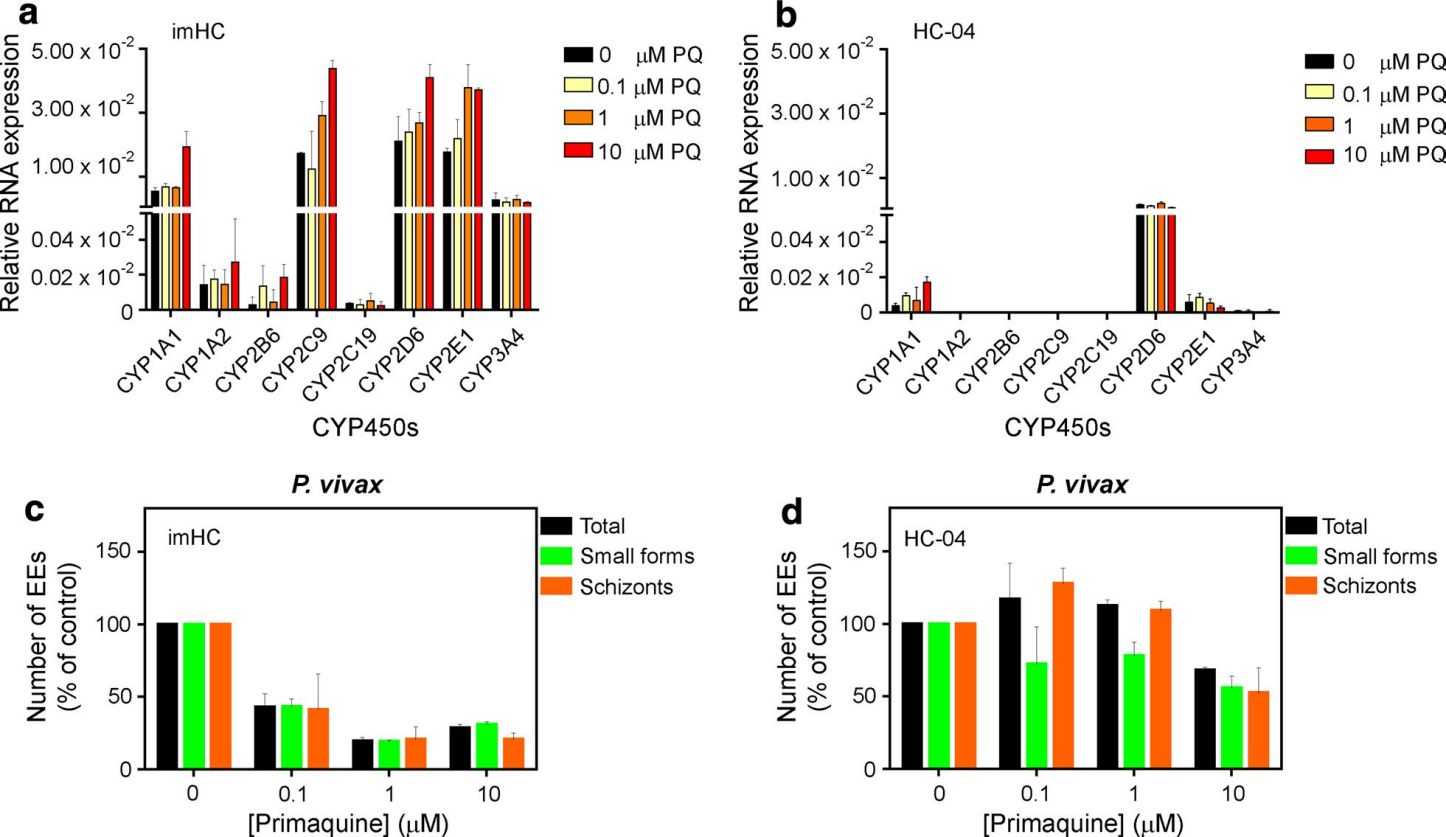
Detection of CYP450s and the inhibitory effect of primaquine on *Plasmodium vivax* liver-stage infection in imHCs. Basal expression levels of CYP450 isotypes were analysed in imHCs (**a**) and HC-04 (**b**) cells using real-time qPCR and are presented as the expression levels relative to those of the house-keeping gene GAPDH. The results are expressed as means ± standard errors of the means of three independent experiments. CYP450 expression levels after a 6-day PQ treatment were also measured in imHCs (**a**) and HC-04 cells (**b**). Hepatocytes were infected with *Plasmodium* sporozoites for 2 h prior to PQ treatment for 6 days. Exoerythocytic forms (EEs) were harvested and visualized by immunofluorescence staining. The number of EEs per well was counted and is shown as a percentage relative to the untreated control. PQ activity against the total, schizont (diameter > 5 µm), and small EE forms (diameter ≤ 5 µm) of *P. vivax* in imHCs (**c**) and HC-04 (**d**) were determined. Representative data from one *P. vivax* isolate are presented as means ± standard errors of the means from an experiment conducted in triplicate

Successful action of a drug such as primaquine required activation by oxidative enzymes [52, 53]. To determine whether imHCs could be used for anti-liver-stage drug screening, the expression of CYP450s in these cells was examined. imHCs had a basal expression of eight major CYP450 isotypes (CYP1A1, CYP1A2, CYP2B6, CYP2C9, CYP2C19, CYP2D6, CYP2E1, CYP3A4), as determined by RT-PCR (Fig. 7a). CYP1A1, CYP2C9, CYP2D6, CYP2E1 and CYP3A4 were expressed at relatively higher levels than CYP1A2, CYP2B6 and CYP2C19 (Fig. 7a). In contrast, only CYP1A1, CYP2D6 and CYP2E1 were expressed in HC-04 cells, and the expression levels were relatively lower than those in imHCs (Fig. 7b).

Even higher expression of six CYP450 isotypes (CYP1A1, CYP1A2, CYP2B6, CYP2C9, CYP2D6, CYP2E1) was induced in imHCs after PQ treatment (Fig. 7a). PQ easily induced the expression of CYP2C9, CYP2D6 and CYP2E1 in a dose–response manner. The expression of CYP1A1, CYP2C9, CYP2D6 and CYP2E1 were significantly upregulated after 10 μM treatment, compared to the untreated controls (*p* < 0.05 in all cases). No significant upregulation was observed in the expression of CYP1A2, CYP2B6, CYP2C19, and CYP3A4 in response to PQ. Among the three CYP450 isotypes in HC-04 cells, the expression of only CYP1A1 was induced by PQ treatment (Fig. 7b). Altogether, imHCs would serve as the site of PQ bioactivation and anti-liver-stage drug activity would be more potent in these cells than in HC-04 cells.

To test PQ sensitivity, hepatocytes were infected with *P. vivax* sporozoites 2 h prior to PQ exposure for a total of 6 days. *Plasmodium vivax*-infected imHCs were sensitive to PQ (Fig. 7c), whereas very low responses to PQ were observed in parasite-infected HC-04 cells (Fig. 7d). In imHCs, PQ activity was detected against schizonts and small forms (presumably hypnozoites), with an IC_50_ value < 0.1 μM for both forms (Fig. 7c). *Plasmodium vivax*-infected HC-04 cells were less responsive to PQ (IC_50_: approximately 10 μM) compared with their imHC counterparts (Fig. 7d). In the liver, PQ is known to produce active metabolites via bioactivation [53]. Therefore, the potent activity of PQ against *P. vivax* parasites in imHCs may be attributed to the higher level of CYP450s in the imHC culture.

## Discussion

Urgent strategies aimed at achieving the current worldwide goal of eradication of malaria have included approaches for discovering drugs that target the *Plasmodium* liver stage, particularly dormant hypnozoites [20]. To date, however, biological features of *P. vivax* hypnozoites remain largely unknown, mainly because of the difficulty of producing large amounts of *P. vivax* sporozoites [42] and the lack of a robust and reliable culture system for this purpose [19]. Currently, humanized liver mouse models appear to be a powerful tool for obtaining human liver-stage parasites in vivo [45, 54–56]. In the context of drug discovery, the cost and technical challenges associated with this animal model render it largely infeasible for early-stage drug screening, although it remains valuable during preclinical testing [56]. Although in vitro primary human hepatocyte models have advanced the ability to identify potential molecules for liver-stage targeting [28, 30, 34], the loss of hepatic functions over time and donor-to-donor variability [28] remains a major challenge when using primary hepatocytes for in vitro assays. Therefore, most studies on liver-stage parasites heavily rely on human hepatoma cell lines as hosts for sporozoite infection [57–59]. However, these hepatoma cells may not be suitable for assessment of drug metabolism and drug interactions because they usually lack various functional CYP450s and other phase I, II, and III drug-metabolizing enzymes. HC-04 cells tend to overgrow in culture, leading to the detachment of host cells, and thus, limiting long-term monitoring of EEs and hindering the establishment of hypnozoites. The reduction in HC-04 cell growth upon reaching confluency might also affect the development of EEs. In addition, infection rates among HC-04 cells alone are relatively low [23] but, as in the present study, a Matrigel co-culture improved this parameter [30]. Infection rates were 4–6 times higher in the Matrgel-HC-04 co-culture than in the original untreated HC-04 model (Patrapuvich R, unpublished data).

To overcome the noted deficiencies of HC-04 cells with respect to supporting liver-stage culture of malaria parasites, a novel hepatocyte cell line, imHC, was therefore tested the ability to support the development of *P. vivax* liver-stage.

imHCs displayed essential liver functions comparable with those of mature hepatocytes [38], ceased proliferation upon reaching a high cell density, and remained in a monolayer even after months of culture. In addition, imHCs expressed the host factors CD81 [48, 60], EphA2 [49] and SR-BI [50], which may be important for sporozoite invasion. No significant difference in infectivity between imHCs (CD81++) and HC-04 (CD81+) cells was observed, suggesting that CD81 was not the primary determinant of *P. vivax* infection. This observation is consistent with the recent study from Manzoni et al. showing that antibodies against SR-BI but not against CD81 inhibit *P. vivax* infection of primary human hepatocyte cultures [61]. The results affirm the potential role of SR-BI during hepatocyte infection by *P. vivax* sporozoites. Manzoni et al. also reported that *P. falciparum,* another major human malarial parasite, relies on CD81 but not SR-BI for host entry [61]. Given these data, it will be interesting to examine whether imHCs can better support *P. falciparum* infection than HC-04 cells. The same study also showed that parasite protein P36 is essential for both SR-BI- and CD81-dependent sporozoite invasion [61]. No direct interaction between P36-dependent invasion and EphA2 has been demonstrated [49, 61, 62]. The role of EphA2 in *P. vivax* infection remains to be further investigated.

In the present study, *P. vivax* infection rate in imHCs (0.14%) was comparable or even higher than that observed in HC-04 cells [23] and in a primary hepatocyte MPCC model [28]. Nevertheless, these infection rates were relatively low in comparison to those observed in in vivo [63, 64]. No significant difference in the development rates of *P. vivax* EEs in imHCs versus those in HC-04 cells based on the percentages of EEs that developed into schizonts (> 10 μm) was observed. However, on day 7, there were many larger EEs among the schizonts growing in imHCs (> 20–45 μm); whereas most EEs in HC-04 cells were smaller (approximately 20–25 μm). The sizes of these day-7 *P. vivax* schizonts in imHCs were similar to those recently reported in MPCC on day 6 (approximately 30 μm) [28] and in a humanized mice model (approximately 40–50 μm) [45]. Larger mature EEs were clearly visible on day 10 (up to approximately 50 μm), but remained smaller than those (60–80 μm) observed in vivo [45]. In imHCs, antibodies specific for PVMs and apicoplasts of EEs revealed a complex cellular structure similar to that observed in vivo [45]. In this study, while MSP-1 expression was not used as a late liver-stage marker for determining parasite maturation, the presence of large multi-nuclei parasites on day 10 confirmed the establishment of mature *P. vivax* schizonts in the imHC culture. Importantly, the long-term culture of *P. vivax*-infected imHCs yielded a population of small (< 10 μm in diameter) forms, presumably hypnozoites, that maintained a single-nuclear structure as detected by histone-acetylation staining of the parasite nuclei [45]. This finding demonstrates the capacity of imHCs to establish *P. vivax* hypnozoites. These presumed hypnozoites in imHCs will continue to require further characterization. It was very interesting to note that a small form observed on day 14 did not exhibit the ‘UIS4-positive prominence’, previously considered as a unique feature of *P. vivax* hypnozoites described by Mikolajczak et al. [45]. In contrast, the young parasite showed on day 4 clearly displayed the prominence pattern. The reliability of this marker as a hypnozoite-specific trait should be considered with care. Here, CSP-VK247 sporozoites produced larger numbers of small forms (77% of EEs) than those of the CSP-VK210 genotype (36% of EEs). This result is in agreement with the recent report in humanized mice model showing that a greater proportion of CSP-VK247 sporozoites (~ 40% of EEs) form hypnozoites compared with CSP-VK210 sporozoites (~ 5% of EEs) [45]. The higher percentage of small EEs obtained in the cultured imHCs compared to infections in in vivo mice may reflect the presence of a sub-population of slow-growing parasites in culture.

The responses of malaria parasites in imHCs to PQ, a drug known to target *Plasmodium* liver stages, were tested to demonstrate the potential use of imHCs as a platform for drug screening. imHCs, which exhibit a cellular physiology closer to that of human hepatocytes compared to HC-04 cells (including CYP450 drug metabolic activity), provide a physiologically relevant platform for drug screening and, thus, would be expected to catch agents that cause drug-induced liver injury [65], particularly during the initial stage of drug discovery. PQ more potently affected EEs in imHCs than in HC-04 cells. Increased PQ sensitivity was associated with higher levels of CYP450 activity in imHCs. CYP2C19, CYP3A4 and CYP2D6 have been identified as the three major enzymes involved in PQ metabolism [53]. The observation that CYP2C19 and CYP3A4 were not induced in imHCs after PQ exposure suggests the essentiality of CYP2D6 in PQ metabolism [52, 66].

Taken together, the findings suggest that imHCs could be an improved hepatoma cell line for study *P. vivax* liver stage parasites. This finding is expected to lead to an array of imHC applications that will facilitate understanding the biology of *P. vivax* hypnozoites and help discover anti-relapse drug, particularly in high-throughput screening formats.

## Conclusions

Previous in vitro cultures of liver-stage malarial parasites have relied on hepatocyte cell lines, particularly the HC-04 line. In short experiments, HC-04 cells support the growth of human malarial parasites *P. falciparum* and *P. vivax*. However, HC-04 cell proliferation is not inhibited by cell–cell contact and host cells detach after a long period. HC-04 cells are not well suitable for long-term hypnozoite cultures that need to study the biology of hypnozoites, identification of putative regulators of parasite dormancy, and drug screening. imHCs, which comprise an immortalized human hepatocyte cell line generated from mesenchymal stem cells, offer an alternative host platform for the culture of human malarial liver stages in vitro. imHCs are superior to HC-04 cells for long-term culture because the former can be maintained for months without overgrowth and detachment, thereby facilitating the development of an in vitro hypnozoite system. imHCs also retain the functions of CYP450s enzymes responsible for drug metabolism and provide a more physiologically relevant model for drug testing. Overall, imHCs potentially serve as a new, robust system for analyzing human *P. vivax* liver-stage parasites, particularly hypnozoites. The resulting models will facilitate a better understanding of the biology of parasites and promote discovery of drugs and vaccines against the liver stage.

## Additional files

**Additional file 1: Figure S1.** Long-term immortalized hepatocyte-like cell (imHC) cultures. Upon achieving confluence, imHCs were maintained in MEM/F12 medium containing 10% fetal bovine serum for more than 6 weeks. Cells were routinely observed, and brightfield images were taken weekly. imHCs remained in a cell monolayer after several weeks of subculture. Scale bar = 50 μm.

**Additional file 2: Figure S2.** Immortalized hepatocyte-like cells (imHCs) were susceptible to *Plasmodium vivax* liver-stage infection. Representative immunofluorescence images depict *P. vivax* exoerythocytic forms (EEs) in imHCs on days 4 and 7 post infection. Anti-UIS4 (red) was used to identify EEs. The hepatocyte nuclei were stained using DAPI. Schizonts and small EEs were observed on day 7 post-infection. Scale bar = 5 μm.

**Additional file 3: Figure S3.** Immortalized hepatocyte-like cells (imHCs) supported *Plasmodium vivax* liver-stage development. Representative immunofluorescence images of *P. vivax* exoerythocytic forms (EEs) in imHCs on day 10 post infection. EEs were visualized using antibodies against parasite UIS4 (red). The hepatocyte nuclei were stained using DAPI. Mature schizonts containing numerous merozoites were observed on day 10 post-infection. Scale bar = 10 μm.

## Abbreviations

BSA: bovine serum albumin
CYP450s: cytochrome P450 isotypes
DAPI: 4′,6-diamidino-2-phenylindole
EEs: exoerythrocytic stages
FBS: fetal bovine serum
IC_50_: 50% inhibitory concentration
iHLCs: induced pluripotent stem cells-derived hepatocyte-like cells
MEM: minimal essential medium
MSCs: mesenchymal stem cells
PBS: phosphate-buffered saline
PQ: primaquine
PVM: parasitophorous vacuole membrane
