# Supplementary material for: A novel immortalized hepatocyte-like cell line (imHC) supports in vitro liver stage development of the human malarial parasite Plasmodium vivax
Source: Malar J. 2018 Jan 25;17:50. doi: 10.1186/s12936-018-2198-4 (PMC5785895; doi:10.1186/s12936-018-2198-4)
Supplement: Supplementary file 1 — Additional file 1: Figure S1. Long-term immortalized hepatocyte-like cell (imHC) cultures. Upon achieving confluence, imHCs were maintained in MEM/F12 medium containing 10% fetal bovine serum for more than 6 weeks. Cells were routinely observed, and brightfield images were taken weekly. imHCs remained in a cell monolayer after several weeks of subculture. Scale bar = 50 μm. [file 12936_2018_2198_MOESM1_ESM.docx]

**Additional Figure S1**

**
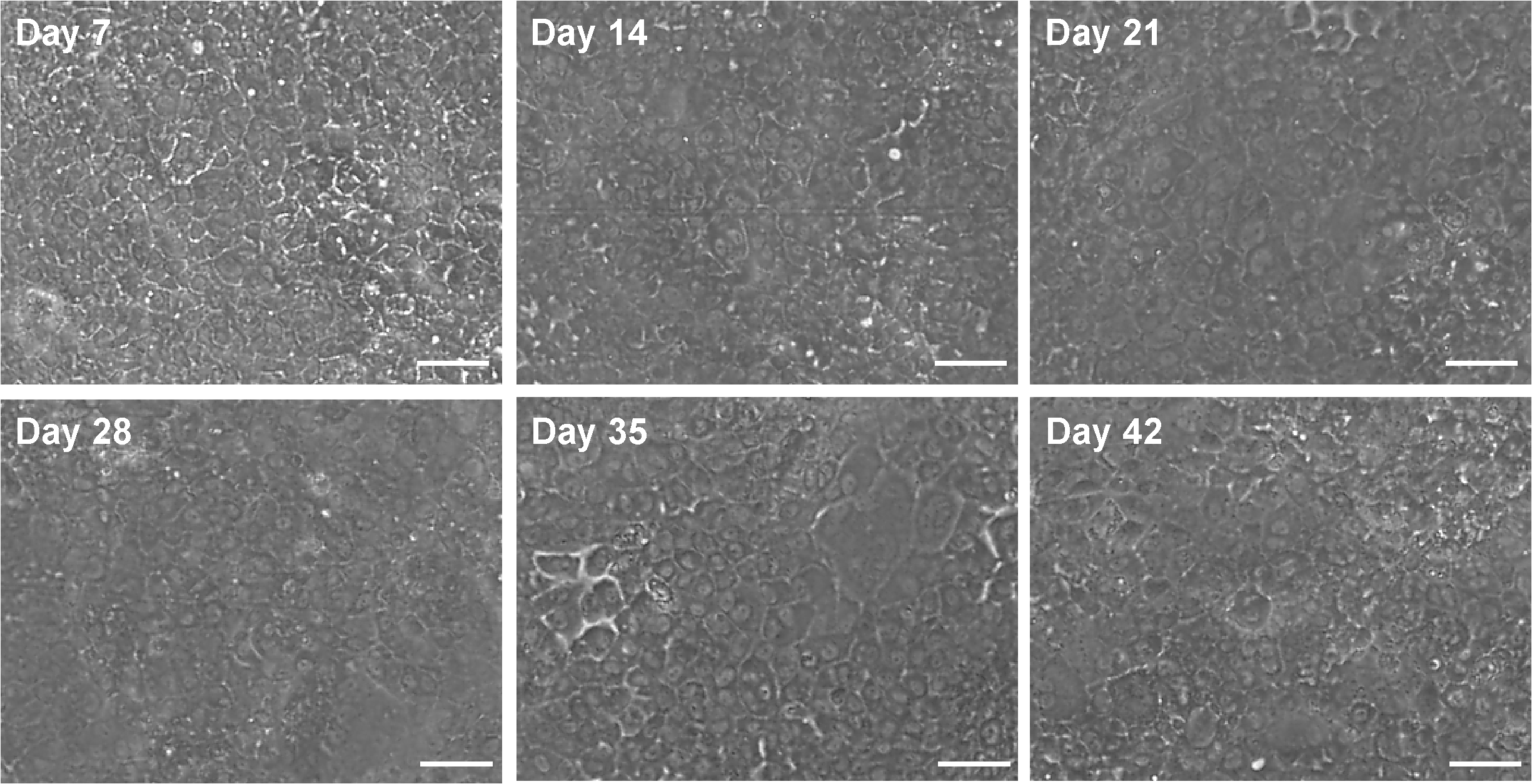
**

**Figure S1. Long-term immortalized hepatocyte-like cell (imHC) cultures.** Upon achieving confluence, imHCs were maintained in MEM/F12 medium containing 10% fetal bovine serum for more than 6 weeks. Cells were routinely observed, and brightfield images were taken weekly. imHCs remained in a cell monolayer after several weeks of subculture. Scale bar = 50 μm.
