# Supplementary material for: A novel immortalized hepatocyte-like cell line (imHC) supports in vitro liver stage development of the human malarial parasite Plasmodium vivax
Source: Malar J. 2018 Jan 25;17:50. doi: 10.1186/s12936-018-2198-4 (PMC5785895; doi:10.1186/s12936-018-2198-4)
Supplement: Supplementary file 2 — Additional file 2: Figure S2. Immortalized hepatocyte-like cells (imHCs) were susceptible to Plasmodium vivax liver-stage infection. Representative immunofluorescence images depict P. vivax exoerythocytic forms (EEs) in imHCs on days 4 and 7 post infection. Anti-UIS4 (red) was used to identify EEs. The hepatocyte nuclei were stained using DAPI. Schizonts and small EEs were observed on day 7 post-infection. Scale bar = 5 μm. [file 12936_2018_2198_MOESM2_ESM.docx]

**Additional Figure S2**

**
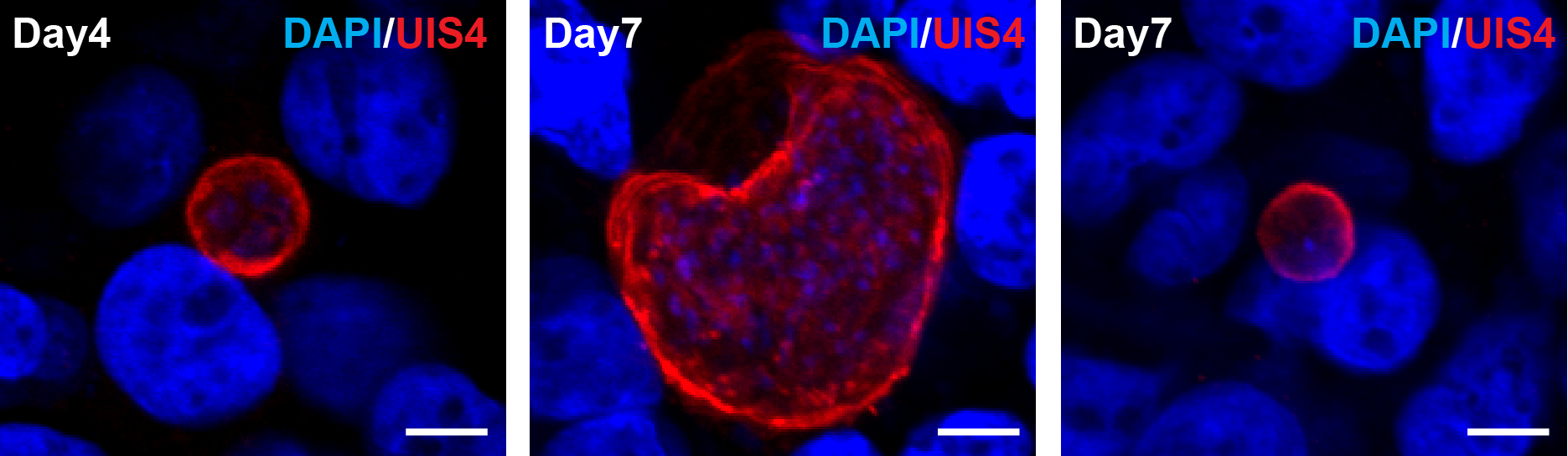
**

**Figure S2. Immortalized hepatocyte-like cells (imHCs) were susceptible to *Plasmodium vivax* liver-stage infection.** Representative immunofluorescence images depict *P. vivax* exoerythocytic forms (EEs) in imHCs on days 4 and 7 post-infection. Anti-UIS4 (red) was used to identify EEs. The hepatocyte nuclei were stained using DAPI. Schizonts and small EEs were observed on day 7 post-infection. Scale bar = 5 μm.
