# Supplementary material for: A novel immortalized hepatocyte-like cell line (imHC) supports in vitro liver stage development of the human malarial parasite Plasmodium vivax
Source: Malar J. 2018 Jan 25;17:50. doi: 10.1186/s12936-018-2198-4 (PMC5785895; doi:10.1186/s12936-018-2198-4)
Supplement: Supplementary file 3 — Additional file 3: Figure S3. Immortalized hepatocyte-like cells (imHCs) supported Plasmodium vivax liver-stage development. Representative immunofluorescence images of P. vivax exoerythocytic forms (EEs) in imHCs on day 10 post infection. EEs were visualized using antibodies against parasite UIS4 (red). The hepatocyte nuclei were stained using DAPI. Mature schizonts containing numerous merozoites were observed on day 10 post-infection. Scale bar = 10 μm. [file 12936_2018_2198_MOESM3_ESM.docx]

**Additional Figure S3**


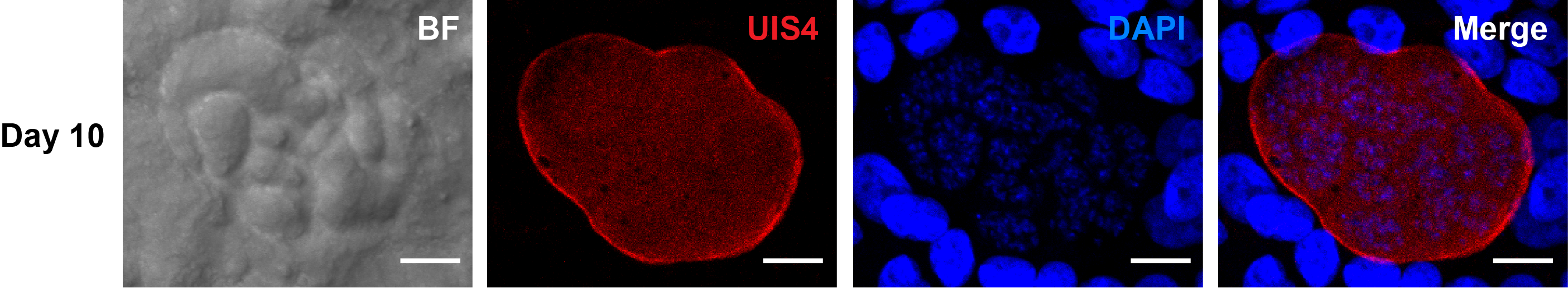


**Figure S3. Immortalized hepatocyte-like cells (imHCs) supported *Plasmodium vivax* liver-stage development.** Representative immunofluorescence images of *P. vivax* exoerythocytic forms (EEs) in imHCs on day 10 post-infection. EEs were visualized using antibodies against parasite UIS4 (red). The hepatocyte nuclei were stained using DAPI. Mature schizonts containing numerous merozoites were observed on day 10 post-infection. Scale bar = 10 μm.
